# The COVID-19 pandemic in the WHO African region: the first year (February 2020 to February 2021)

**DOI:** 10.1017/S0950268821002429

**Published:** 2021-11-04

**Authors:** Benido Impouma, Franck Mboussou, Bridget Farham, Caitlin M. Wolfe, Krys Johnson, Catherine Clary, Richard Mihigo, Ngoy Nsenga, Ambrose Talisuna, Zabulon Yoti, Antoine Flahault, Olivia Keiser, Abdou Salam Gueye, Joseph Cabore, Matshidiso Moeti

**Affiliations:** 1 World Health Organization, Regional Office for Africa, Brazzaville, Congo; 2 Institute of Global Health, University of Geneva, Geneva, Switzerland; 3 College of Public Health, University of South Florida, Tampa, Florida, USA; 4 Department of Epidemiology and Biostatistics, Temple University, Philadelphia, Pennsylvania, USA

**Keywords:** African region, COVID-19, pandemic response, public health and social measures

## Abstract

The World Health Organization African region recorded its first laboratory-confirmed coronavirus disease-2019 (COVID-19) cases on 25 February 2020. Two months later, all the 47 countries of the region were affected. The first anniversary of the pandemic occurred in a changed context with the emergence of new variants of concern (VOC) and growing COVID-19 fatigue. This study describes the epidemiological trajectory of COVID-19 in the region, summarises public health and social measures (PHSM) implemented and discusses their impact on the pandemic trajectory. As of 24 February 2021, the African region accounted for 2.5% of cases and 2.9% of deaths reported globally. Of the 13 countries that submitted detailed line listing of cases, the proportion of cases with at least one co-morbid condition was estimated at 3.3% of all cases. Hypertension, diabetes and human immunodeficiency virus (HIV) infection were the most common comorbid conditions, accounting for 11.1%, 7.1% and 5.0% of cases with comorbidities, respectively. Overall, the case fatality ratio (CFR) in patients with comorbid conditions was higher than in patients without comorbid conditions: 5.5% *vs.* 1.0% (*P* < 0.0001). Countries started to implement lockdown measures in early March 2020. This contributed to slow the spread of the pandemic at the early stage while the gradual ease of lockdowns from 20 April 2020 resulted in an upsurge. The second wave of the pandemic, which started in November 2020, coincided with the emergence of the new variants of concern. Only 0.08% of the population from six countries received at least one dose of the COVID-19 vaccine. It is critical to not only learn from the past 12 months to improve the effectiveness of the current response but also to start preparing the health systems for subsequent waves of the current pandemic and future pandemics.

## Key results

- On 24 February 2021, 12 months after the notification of the first laboratory-confirmed COVID-19 case, the African region recorded a total of 2 795 424 confirmed cases (2.5% of cases reported globally) including 74 841 deaths [case fatality ratio (CFR) of 2.7%].
- The pandemic trajectory showed two waves: the first one from 25 February 2020 to 31 October 2020 with a peak in late July and the second wave from 01 November 2020 to 24 February 2021 with a peak in early January 2020.
- The second wave of the pandemic coincided with the emergence of new variants of concern.
- Hypertension, diabetes and human immunodeficiency virus (HIV) infection were the most common comorbid conditions, accounting for 11.1%, 7.1% and 5.0%, respectively
- During the study period, only 0.08% of the population from six countries received at least one dose of the COVID-19 vaccine.

In December 2019, a novel strain of coronavirus emerged among humans in Wuhan, China and was identified as severe acute respiratory syndrome coronavirus 2 (SARS-CoV-2) [1], which causes coronavirus disease-2019 (COVID-19). There is a wide range of symptoms among positive cases, from mild flu-like symptoms, to severe illness, usually characterised by respiratory distress. By the end of January 2020, COVID-19 was responsible for over 200 deaths and 9800 cases globally and was declared a Public Health Emergency of International Concern by the World Health Organization (WHO) [1]. On 11 March 2020, the WHO declared the COVID-19 a pandemic [1] considering its alarming spread and severity as most WHO regions reported confirmed cases.

The WHO is grouped into six regions: Western Pacific, Europe, South-East Asia, Eastern Mediterranean, the Americas and the African region [2]. The WHO African region (‘subsequently referred to as the African region’) is made up of 47 countries, which are all part of the African continent. Seven additional countries of the African continent belong to the WHO Eastern Mediterranean region [3]. The African continent in general and the African region, in particular, is characterised by a large infectious disease burden and weak public health infrastructure [4], posing a threat to global health security [5]. The first laboratory-confirmed case in the African region was reported on 25 February 2020, in Algeria, in an individual who had travelled from Milan, Italy [6]. Cases were then detected in Nigeria on 28 February 2020 and Senegal on 2 March 2020 in travellers [6]. On 13 May 2020, all the 47 countries of the African region were affected, with Lesotho being the last to report laboratory-confirmed cases [7]. On 24 February 2021, it was 1 year since the first COVID-19 case was confirmed in the African region. By then, the context of the COVID-19 response had changed with the emergence of variants of concern (VOC), the availability of vaccines as a new response tool and growing COVID-19 fatigue resulting in reduced adherence to public health and social measures (PHSM) [8].

As we enter the second year of the pandemic, it is crucial to assess the COVID-19 epidemiological situation and progress made in implementing response measures in order to incorporate lessons learned. In this paper, we describe the epidemiological evolution of COVID-19 in the African region, the preparedness interventions preceding the confirmation of the first case and summarise public health measures implemented by national authorities. Furthermore, we reviewed the lessons learned during the first year of the COVID-19 pandemic and discuss their implications on the ongoing pandemic and future outbreaks.

We used the regional line list of COVID-19 laboratory-confirmed cases maintained by the WHO Regional Office for Africa (WHO/AFRO) for our analysis. This line list contains data on COVID-19 laboratory-confirmed cases reported to WHO/AFRO by member states as per their obligations under the International Health Regulations (2005) [9] and the Integrated Disease Surveillance and Response Strategy (IDSR) [10]. Only data from the 47 member states of the African region are included in this line list. The seven countries belonging to the WHO Eastern Mediterranean region (Tunisia, Morocco, Egypt, Somalia, Sudan, Libya and Djibouti) were not included in the analysis.

Only cases meeting the COVID-19 case definition in line with the most recently updated WHO technical guidance [11] and which were laboratory confirmed using a reverse transcriptase-polymerase chain reaction (RT-PCR) test or an antigen rapid diagnostic test approved by the WHO and officially reported by member states, were included in the regional line list. We computed the cumulative number of cases, deaths and health worker infections by country as well as the case fatality ratio (CFR) (cumulative number of deaths divided by the cumulative number of cases, as a percentage) and the attack rate per million population (cumulative number of cases divided by the total population). Population estimates for each member state were obtained from the World Bank data [12]. COVID-19 confirmed cases that were still alive and not yet discharged at the date of the review were considered as active cases.

We used the dataset on SARS-CoV-2 vaccination maintained by the WHO/AFRO to generate the numbers of people vaccinated by at least one dose of the COVID-19 vaccine. All the vaccines received and administrated by member states were included in this dataset irrespective of their origin [bilateral cooperation, COVID-19 Vaccines Global Access (COVAX), African Vaccine Acquisition Trust (AVAT)].

For the analysis of age, gender and comorbid conditions, we merged the latest available country-specific line lists of COVID-19 confirmed cases shared with WHO/AFRO by 13 member states: Burkina Faso, Chad, Cote d'Ivoire, Democratic Republic of Congo (DRC), Eswatini, Guinea, Namibia, Niger, Rwanda, Senegal, Seychelles, Sao Tome and Principe and Uganda. A total of 233 261 confirmed cases were recorded in the merged line list.

Data were analysed using R version 4.0.3 [13] for statistical analysis.

On 24 February 2021, 12 months after the notification of the first laboratory-confirmed COVID-19 case, the African region recorded a total of 2 795 424 confirmed cases (2.5% of cases reported globally), 74 841 deaths (2.9% of global deaths) and 2 542 895 recoveries (91% of all cases). The CFR was estimated at 2.7%. The number of active cases was 177 688 in 46 out of 47 countries; this figure does not include the United Republic of Tanzania, which stopped reporting COVID-19 cases to WHO/AFRO on 07 May 2020.

South Africa (1 507 448 confirmed cases; 53.9% of all cases in the region), Ethiopia (155 234; 5.6%), Nigeria (153 842; 5.5%), Algeria (112 461; 4.0%) and Kenya (104 780; 3.7%) were the five countries with the highest number of cumulative confirmed cases. These five countries accounted for 72.7% of all cases in the African region.

Cape Verde (2757 cases/100 000 population), Seychelles (2629 cases/100 000), South Africa (2574 cases/100 000), Namibia (1532 cases/100 000) and Eswatini (1472 cases/100 000) recorded the highest COVID-19 cumulative attack rate (number of cases per 100 000 population) in the African region. Four of these five countries (South Africa excluded) with the highest attack rate have a population of less than 2.5 million inhabitants.

South Africa (51 560 deaths; 68.7% of all deaths), Algeria (3069; 4.1%), Ethiopia (2718; 3.6%), Kenya (2066; 2.8%) and Nigeria (2031; 2.7%) recorded the highest number of deaths in the African region. These five countries accounted for 81.9% of all deaths. The following 13 countries recorded CFRs higher than the overall CFR in the African region (2.7%): Mali (4.4%), Liberia (4.2%), Zimbabwe (4.1%), Comoros (4.1%), Eswatini (3.9%), Niger (3.7%), Chad (3.7%), Malawi (3.7%), South Africa (3.4%), Gambia (3.0%), Lesotho (2.9%), Senegal (2.9%) and the Democratic Republic of the Congo (2.9%).

The cumulative number of health worker infections was 101 885, representing 3.6% of all COVID-19 confirmed cases. Five countries recorded more than 10% of cases among health workers out of all cases: Guinea-Bissau (11.7%), Zimbabwe (11.7%), Liberia (11.1%), Algeria (10.6%) and Seychelles (10.4%).

**Fig. 1.**
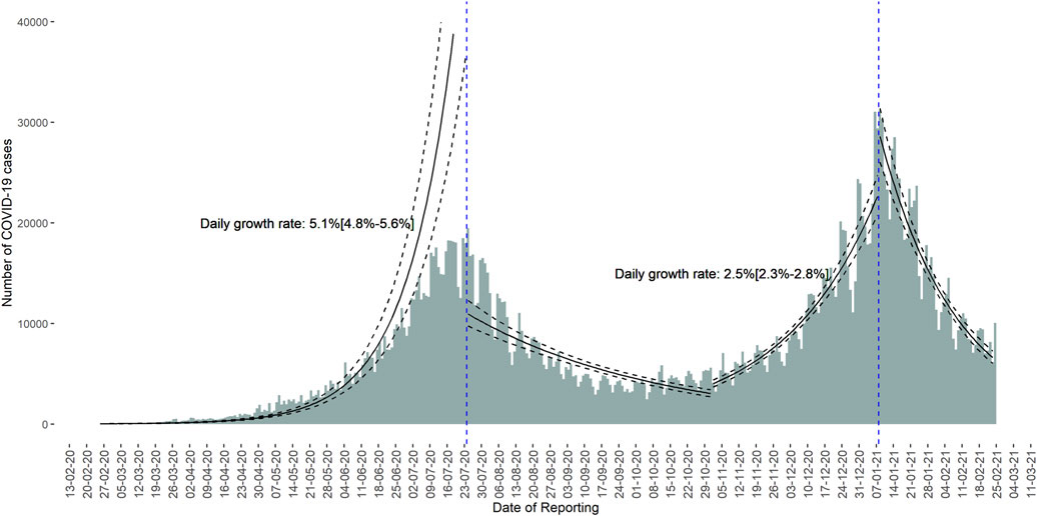
Distribution of COVID-19 cases by the reporting date and log-linear fit in the African region (data as of 24 February 2021).

Figure 1 presents the daily distribution of COVID-19 cases from 25 February 2020 to 24 February 2021 and log-linear fit. Following a slow increase from 25 February 2020 to 01 May 2020, new cases reported increased exponentially and peaked on 24 July 2020 (19 478 cases reported). This first peak was followed by 8 weeks of decline and then a 6-week plateau. An upsurge in new cases reported was seen from 1 November 2020, which peaked on 6 January 2021 (31 049 cases reported) before declining. The pandemic trajectory showed two waves: the first wave from 25 February 2020 to 31 October 2020 with a peak in late July and the second wave from 1 November 2020 to 24 February 2021 with a peak in early January 2021.

As shown in Figure 1, the daily growth rate was 5.1% (4.8%; 5.6%) in the increasing phase of the first wave compared to 2.5% (2.3%; 2.8%) in the increasing phase of the second wave.

From 25 February 2020 to 1 May 2020 (period 1), 46 countries out of 47 in the African region reported at least one COVID-19 confirmed case; Lesotho being the only country that did not report cases during this period. South Africa, Algeria and Nigeria reported the highest number of cases during period 1, accounting respectively for 21.9% (*n* = 5951 out of 27 196), 15.3% (*n* = 4154) and 8.0% (*n* = 2170) of all cases. These three countries recorded the highest number of international inbound tourists in 2019 [14]. In a study assessing the risk of importation of COVID-19 cases in Africa conducted in early February 2020 [15], South Africa and Algeria were among the countries with the highest risk while Nigeria was among countries with the second-highest importation risk ranking. During period 1, Equatorial Guinea (323 cases per million population), Mauritius (262 cases per million) and Cape Verde (223 cases per million) recorded the highest attack rate. All these countries are island nations with less than 2.5 million inhabitants. This early stage of the pandemic was marked by a high CFR in most countries with the healthcare system not well prepared for early detection of cases and case management: median of 4.85 ranging from 0% to 55.6%. Five countries did not record COVID-19 related deaths during period 1: Seychelles, Eritrea, Namibia, Uganda and Comoros.

From 2 May 2020 to the peak of the first wave of the pandemic in the African region which occurred on 24 July 2020 (period 2), South Africa reported 63.6% of all cases (*n* = 416 045 out of 654 028) followed by Nigeria (5.7%; *n* = 37 369) and Ghana (4.7%; *n* = 30 895). All the 47 countries of the African region reported COVID-19 confirmed cases during period 2. From April 2021, the pandemic pattern started to shift from sporadic cases (most cases imported) to community transmission [16]. During this period, South Africa also recorded the highest attack rate (7104.8 cases per million) followed by two island nations with small population: Sao Tome and Principe (3915. 3 cases per million) and Cape Verde (3813.2 cases per million). By the end of period 2, Eritrea and Seychelles were the only countries that did not record COVID-19 related deaths by the end of period 2. The median CFR was 2.0% ranging from 0% to 12.3%.

The third period, from 25 July to 31 October corresponded to the decreasing phase of the first wave of the pandemic and had the same characteristics as the second period in terms of countries reporting the highest cases and attack rate, as well as median CFR. Seychelles and Eritrea did not report deaths during this period.

The period 4, from 01 November 2020 to 24 February 2021 corresponded to the second wave of the pandemic. During this period, South Africa reported 52.9% of cases (*n* = 781 996 out of 1.47 million) followed by Nigeria (6.1%; *n* = 90 989) and Zambia (4.1%; *n* = 60 052). Seychelles (24 665.8 cases per million), South Africa (13 354.2 cases per million) and Cape Verde (11 579.6 recorded the highest attack rate. Seychelles and Eritrea reported 14 deaths out of 2408 cases and seven deaths out of 2326 deaths during this period, respectively. The median CFR was 1.3%, ranging from 0% to 5.0%.

On 24 February 2021, the pandemic trajectory showed a downward trend or plateau in 35 countries out of 46 (76.1%) and an upward trend in 11 countries (23.9%). The following countries experienced an upsurge of cases in the last 4 weeks (28 January 2021–24 February 2021): Benin, Burundi, Cameroon, Chad, Equatorial Guinea, Ethiopia, Gabon, Guinea, Madagascar, Namibia and South Sudan. The epidemic trajectory showed fluctuations or plateau in the last 4 weeks (28 January 2021–24 February 2021) in 11 other countries: Botswana, DRC, Ghana, Guinea-Bissau, Kenya, Liberia, Mauritius, Mozambique, Sao Tome and Principe, Senegal and Seychelles. The 24 remaining countries in the African region experienced a downward trend during the 4-week period preceding the end of the first year of the pandemic. The heterogenicity of the pandemic across countries may have been influenced by restrictions imposed by governments at the early stage of the pandemic as well as testing policies. Table 1 summarises government restrictions established in the first 2 months of the pandemic (March–April 2021) and testing policy in terms of the population targeted for testing at the end of the first year into the pandemic.

**Table 1.** Government restrictions measures established at the early stage of the pandemic and testing policies in the WHO African region

Country	All International flights suspended in the first 2 months (March–April 2020)	Lockdown in the first 2 months(March–April 2020)	Testing policy (population targeted for testing) in February 2021
Algeria	Yes	Full lockdown	Symptomatic cases
Angola	Yes	Partial lockdown	Symptomatic cases
Benin	Yes	No lockdown	Symptomatic cases
Botswana	Yes	Partial lockdown	Symptomatic cases
Burkina Faso	Yes	No lockdown	Open public test
Burundi	Yes	No lockdown	Symptom & key groups
Cameroon	Yes	No lockdown	Symptom & key groups
Cape Verde	Yes	No lockdown	Open public test
Central African Republic	Yes	No lockdown	Symptom & key groups
Chad	Yes	No lockdown	Symptomatic cases
Comoros	Yes	No lockdown	Symptomatic cases
Congo	Yes	Partial lockdown	Symptomatic cases
Cote d'Ivoire	Yes	Partial lockdown	Symptomatic cases
Democratic Republic of the Congo	Yes	Partial lockdown	Symptom & key groups
Equatorial Guinea	Yes	Partial lockdown	Symptomatic cases
Eritrea	Yes	Partial lockdown	Open public test
Eswatini	Yes	Partial lockdown	Symptomatic cases
Ethiopia	No	No lockdown	Open public test
Gabon	Yes	Partial lockdown	Open public test
Gambia	Yes	No lockdown	Open public test
Ghana	Yes	Partial lockdown	Symptomatic cases
Guinea	Yes	No lockdown	Open public test
Guinea-Bissau	Yes	No lockdown	Symptomatic cases
Kenya	Yes	No lockdown	Symptomatic cases
Lesotho	Yes	Partial lockdown	Symptom & key groups
Liberia	Yes	Partial lockdown	Open public test
Madagascar	Yes	Partial lockdown	Symptom & key groups
Malawi	Yes	Partial lockdown	Symptomatic cases
Mali	Yes	No lockdown	Symptomatic cases
Mauritania	Yes	No lockdown	Open public test
Mauritius	No	Partial lockdown	Open public test
Mozambique	Yes	No lockdown	Symptom & key groups
Namibia	Yes	Partial lockdown	Symptomatic cases
Niger	Yes	No lockdown	Open public test
Nigeria	Yes	Partial lockdown	Symptom & key groups
Rwanda	Yes	Partial lockdown	Symptomatic cases
Sao Tome and Principe	No	No lockdown	Symptomatic cases
Senegal	Yes	No lockdown	Open public test
Seychelles	Yes	Partial lockdown	Open public test
Sierra Leone	Yes	Partial lockdown	Open public test
South Africa	Yes	Full lockdown	Open public test
South Sudan	Yes	No lockdown	Symptomatic cases
Togo	Yes	Partial lockdown	Symptomatic cases
Uganda	Yes	Partial lockdown	Symptom & key groups
United Republic of Tanzania	Yes	No lockdown	Not available
Zambia	Yes	No lockdown	Symptom & key groups
Zimbabwe	Yes	Partial lockdown	Open public test

**Fig. 2.**
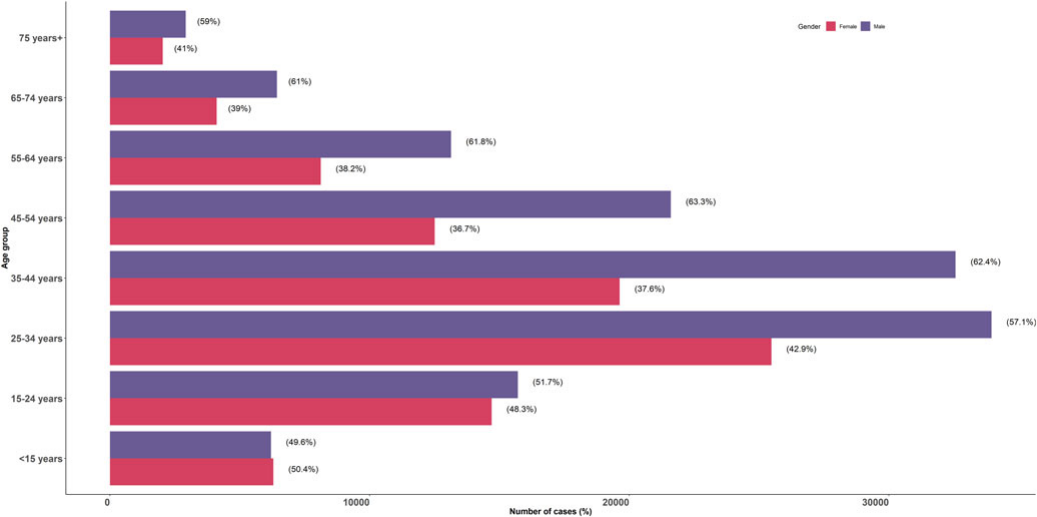
Distribution of COVID-19 cases from 13 countries of the WHO African region by age group and gender.

Out of the 233 261 cases from 13 countries that shared detailed line lists, 139 257 were male (59.7%) and 90 004 were female (40.3%). Most (91.4%) cases were aged below 65 years (*n* = 213 268), with 6.8% aged 65 years or above (*n* = 15 802) and 1.8% cases missing data on age (*n* = 4 191). The distribution of COVID-19 cases by age group and gender is illustrated in Figure 2. There were more males than females among COVID-19 cases in all age groups.

Table 2 presents the distribution of cases by gender, age group and presence of symptoms for each of the 13 countries. The proportion of asymptomatic cases ranged from 50.9% to 89.6%.

**Table 2.** Distribution of COVID-19 cases from 13 African countries by gender, age groups and presence of symptoms

Country	Data as of	Cumulative cases	Gender		Age groups			% of cases by age group			Deaths in cases aged 65 years+		Deaths in cases aged below 65 years		Asymptomatic cases	
			Female	% Female	below 65 years	65 years+	Age missing	65 years+	below 65 years	Age missing	# deaths	CFR	# deaths	CFR	# cases	% of all cases
Burkina Faso		8203	3464	42.2	7631	570	2	6.9	93	0.1	38	6.7	34	0.4	5724	69.8
Chad	31/01/2021	4779	1302	27.2	4084	293	402	6.1	85.5	8.4	46	15.7	78	1.9	4069	85.1
Cote d'Ivoire	24/02/2021	32 440	13 884	42.8	30 819	1265	356	3.9	95	1.1	89	7	99	0.3	29 078	89.6
DRC	24/02/2021	25 556	9007	35.2	22 662	2059	835	8.1	88.7	3.2	231	11.2	378	1.7	22 119	86.6
Eswatini	13/02/2021	16 488	8581	52	15 244	1209	35	7.3	92.5	0.2	53	4.4	61	0.4	13 130	79.6
Guinea	24/02/2021	19 074	6395	33.5	17 825	1247	2	6.5	93.5	0	50	4	47	0.3	Not available	
Namibia	15/01/2021	29 244	14 377	49.2	28 197	1018	29	3.5	96.4	0.1	82	8.1	157	0.6	14 889	50.9
Niger	24/02/2021	4160	1258	30.2	3584	362	214	8.7	86.2	5.1	75	20.7	91	2.5	3417	82.1
Rwanda	24/02/2021	18 443	6066	32.9	17 453	839	151	4.5	94.6	0.9	112	13.3	144	0.8	Not available	
Sao Tome & Principe	06/02/2021	2057	1014	49.3	1873	170	14	8.3	91.1	0.6	8	4.7	20	1.1	Not available	
Senegal	24/02/2021	29 066	11 948	41.1	23 092	4662	1312	16.0	79.4	4.6	117	2.5	73	0.3	Not available	
Seychelles	24/02/2021	3451	1675	48.5	2510	102	839	3.0	72.7	24.3	8	7.8	3	0.1	Not available	
Uganda	24/02/2021	40 300	15 033	37.3	38 294	2006	0	5.0	95.0	0.0	133	6.6	203	0.5	Not available	
Total		233 261	94 004	40.3	213 268	15 802	4191	6.8	91.4	0.0	1042	6.6	1388	0.7		

As shown in Table 3, 7685 had at least one comorbid condition (3.3% of all cases). Hypertension, diabetes and human immunodeficiency virus (HIV) infection were the most common comorbid conditions, accounting respectively for 11.1%, 7.1and 5.0% of cases with comorbidities. The proportion of cases with diabetes among COVID-19 confirmed cases was lower than in general population: 0.26% against a median of 5.0% (range: 2.6%; 14.2%) [17]. However, the proportion of cases with HIV among COVID-19 cases was not significantly different to that of the general population: 0.16% against a median of 0.068% (range: 0.001; 0.97) [18]. The highest CFR was recorded in patients with obesity (36.4%), tuberculosis (21.4%), chronic lung disease (21.1%), renal failure (18.8%) and neurological disease (15.4%). Overall, the CFR in patients with comorbid conditions was higher than in patients without comorbid conditions: 5.5% *vs.* 1.0% (*P* < 0.0001).

**Table 3.** Distribution of COVID-19 cases with comorbid conditions by type of comorbidity (data as of 24 February 2021 from 13 African countries)

Comorbid conditions	Asymptomatic	Symptomatic	Total cases	% of cases with comobid conditions	% of all confirmed cases	% symptomatic	Number of deaths	CFR
Hypertension	425	428	853	11.1	0.37	50.2	104	12.2
Diabetes	346	262	608	7.9	0.26	43.1	62	10.2
HIV	129	253	382	5.0	0.16	66.1	9	2.4
Cardiovascular disease	73	303	376	4.9	0.16	80.6	30	8.0
Asthma	153	98	251	3.3	0.11	39.0	14	5.6
Pregnancy	41	17	58	0.8	0.02	29.3	1	1.7
Renal failure	20	28	48	0.6	0.02	58.3	9	18.8
Chronic lung disease	8	30	38	0.5	0.02	78.9	8	21.1
neurological disease	1	25	26	0.3	0.01	96.2	4	15.4
Drepanocytosis	13	4	17	0.2	0.01	23.5	1	5.9
Tuberculosis	4	10	14	0.2	0.01	71.4	3	21.4
Cancer	6	10	16	0.2	0.01	53.8	2	15.4
Cerebrovascular disease	11	1	12	0.2	0.01	8.3	0	0.0
Obesity	6	5	11	0.1	0.0	45.5	4	36.4
Gout	2	2	4	0.1	0.0	50.0	0	0.0
Hepatitis	0	1	1	0.0	0.0	100.0	0	0.0
Other	3397	1573	4970	64.7	2.13	31.6	166	3.3
Total	4635	3050	7685	100.0	3.29	37.9	420	5.5

As of 24 February 2021, 25 904 273 SARS-CoV-2 tests (molecular and antigen) were performed in the African region (data available for 43 countries out of 47), representing 241.5 tests per 10 000 population. Botswana (3289 tests per 10 000 population), Mauritius (2774 tests/10 000), Gabon (2413 tests/10 000), Cape Verde (2212 tests/10 000) and South Africa (1546 tests/10 000) recorded the highest testing performance. For an effective surveillance system, the testing performance is expected to surpass 10 tests per 10 000 population per week [19]. Out of the 43 countries assessed based on available data, only 18 (41.8%) surpassed this threshold in the last 4 weeks (28 January- 24 February 2021).

Although it was predicted that Africa would suffer a massive loss of lives due to this pandemic, the number of COVID-19 cases and deaths has been relatively low across the African region [20]. Several authors have asserted that the number of cases reported in Africa does not reflect the true burden of the pandemic as many COVID-19 infections are presumed to remain undetected [21–23]. Mulenga *et al*. [22], conducted a cross-sectional, cluster-sample survey of households in six districts of Zambia in July 2020 and estimated that 454 708 SARS-CoV-2 infections [95% confidence interval (CI) 312 705–596 713] occurred in the six districts between March and July 2020, compared with 4917 laboratory-confirmed cases reported in official statistics from the Zambia National Public Health Institute. This means that only one laboratory-confirmed case was reported for every 92 SARS-CoV-2 infections that occurred in the community [21]. The low case detection rate in Africa is mainly due to (i) low testing capacity with only a few countries continuously surpassing the threshold of 10 tests performed per 10 000 population every week (ii) testing strategies focusing mostly on people meeting the clinical case definition, while most people infected have no or mild symptoms [21]. In the study conducted by Mulenga *et al*. in Zambia [22], most of the cases identified were asymptomatic or had mild symptoms. The high promotion of asymptomatic cases among COVID-19 cases in Africa may be due to the large proportion of young people in most countries, with less severe disease presentation and relatively low rates of comorbid conditions in comparison to many Western countries [23, 24]. Younger population, lower life expectancy, low rates of comorbid conditions and possible cross-immunity are also among proposed explanations for this relatively low mortality [24, 25]. However, the African COVID-19 Critical Care Outcomes Study (ACCCOS) Investigators found that mortality in critically ill patients with COVID-19 is higher in African countries than reported from studies done in Asia, Europe, North America and South America [26]. In a prospective systematic postmortem surveillance study, Mwananyanda *et al*. [27] found COVID-19 infections both in deaths that occurred in the community and in people who died in hospital, suggesting that COVID-19-related deaths are also under-detected although to a lesser extent than COVID-19 cases.

The COVID-19 pandemic in the African region has been heterogeneous over time across countries. At the early stage of the pandemic, countries with the highest volume of air travel such as South Africa, Algeria and Nigeria, were among the most affected in terms of the number of confirmed cases reported. Once community transmission was established, the high burden countries varied from one period to another depending on preventive measures applied by the governments and on testing capacities and strategies. South Africa remained the most affected country, accounting for 53.9% of cumulative confirmed cases reported in the region. However, island countries with low population sizes reported the highest attack rate in the African region.

During the first year, the African region was hit by two waves of the pandemic. The daily growth rate in the ascending phase of the second wave was lower than in the first wave. This may be due to lower incidence at the beginning of the first wave compared to the beginning of the second wave, resulting in greater uncertainty. However, the trajectory of the second wave was steeper and higher than that of the first wave. Indeed, the second wave peaked after 66 days against 150 days for the first wave and the second wave peak was 1.5 times higher than that of the first wave.

Data from countries that shared detailed line lists showed that most cases recorded were asymptomatic. This may explain the issue of low detection rate raised by several authors [20, 22, 28]. Countries in Africa need to adjust their testing strategies, which are still targeting people meeting the COVID-19 clinical case definition. A community-based approach using active case search around households of new confirmed cases could help to improve the detection rate as well as better documenting community clusters, thus contributing to breaking chains of transmission.

As the pandemic unfolded in China and subsequently in Europe and the United States, the WHO, as the directing and coordinating authority on international health within the United Nations System [29], developed a series of technical guidance documents [30] covering 14 topics (animal-human interface and food safety; clinical care; critical preparedness, readiness and responses; essential health services; essential resource planning; infection prevention and control/water, sanitation and hygiene; laboratory and diagnosis; mass gatherings; risk communication and community engagement; schools, businesses and institutions; scientific briefs; surveillance, case investigation and epidemiological protocols; travel, points of entry and border health; vaccines; and vulnerable population and fragile settings). These documents aimed to provide technical guidance to member states on the implementation of the different pillars of the COVID-19 preparedness and response. WHO/AFRO specifically conducted 53 webinars to build country capacities on the use of these technical guidelines.

Building on past experiences in responding to threats arising from epidemic and pandemic prone diseases, member states and the WHO activated their emergency response procedures in line with the Emergency Response Framework [31] and the Integrated Diseases Surveillance and Response (IDSR) strategy [10]. This resulted in the rapid implementation of multisectoral coordination mechanisms and structures at regional and national levels, headed at regional level by the Regional Director of WHO/AFRO and at national level by either Heads of State or Prime Ministers, with technical taskforces established to guide evidence-based decision-making.

Further, following the release of the global Strategic Preparedness and Response Plan (SPRP) [32] by WHO in early February 2020, WHO/AFRO developed and released an SPRP specific for the African region [33], detailing the targeted needs and goals across all response pillars. Several other initiatives aiming at supporting the response against the COVID-19 pandemic in Africa were launched by other partners such as the World Bank [34], the African Development Bank [35], the Africa Centre for Disease Control and Prevention [36] and the Global Fund to fight AIDS, Tuberculosis and Malaria [37].

As reported by several authors [28, 38–40], the initial rapid upsurge in the number of cases and deaths in all 47 countries in the African region within the first few months of the pandemic triggered the implementation of PHSM by member states.

There are added concerns surrounding the new VOC that emerged in South Africa and the United Kingdom. As of 24 February 2021, the Beta variant initially identified in South Africa had been detected in seven other countries (Botswana, Comoros, Ghana, Kenya, Mozambique, Zambia and Zimbabwe) [41], while the Alpha variant, initially identified in the United Kingdom had been detected in six countries (the Democratic Republic of the Congo, Ghana, Gambia, Nigeria, Senegal and South Africa). The Beta variant was known to be predominant in South Africa, driving the second wave of the pandemic. The emergence of these variants underscores the need for strengthening genomic surveillance and collaboration between countries given the limited number of countries with the capacity to perform genomic surveillance.

Vaccine procurement, delivery and rollout across the African region has been slow and patchy. While vaccination coverage has increased in wealthier nations, so far few countries in the African region have received vaccines (i) through COVAX, a global risk-sharing mechanism for pooled procurement and equitable distribution of COVID-19 vaccines, co-led by the Coalition for Epidemic Preparedness Innovations (CEPI), Gavi and the WHO, alongside UNICEF as key delivery partners [42], (ii) AVAT, a mechanism aiming to ensure widespread access to COVID-19 vaccines across Africa set up by the African Union [36], (iii) or procured vaccines through bilateral agreements. Most countries that have received vaccines have started their rollout. Further delays in COVID-19 vaccination rollout, resulting in no small part from gross inequality of access to vaccine supply in poorer countries, will allow for a continued climb of case counts across the region, particularly when combined with pandemic response fatigue. As of 24 February 2021, a total of 854 799 people had received at least one dose of COVID-19 vaccine in the African region, representing 0.08% of the population compared to 8.1% in North America, 5.6% in Europe and 2.3% in South America [43]. Only Six countries have launched their vaccination campaigns: Algeria (75 000 people received at least one dose of vaccine; 0.17% of the population, vaccination launched on 29 January 2021), Mauritius (3843, 0.3%, vaccination launched on 26 January 2021), Senegal (4087; 0.02%, vaccination launched on 23 February 2021), Seychelles (47 188; 47.9%, vaccination launched on 10 January 2021), South Africa (41 809; 0.07%, vaccination launched on 17 February 2021) and Zimbabwe (7872; 0.05%, vaccination launched on 22 February 2021).

For the first time since the establishment of the WHO Emergency Framework in 2013 [31] which set out WHO's commitment to health emergency response, all 47 member states have been affected simultaneously by an outbreak, challenging the capacity of WHO/AFRO and other partners to provide the required support. The deployment of technical experts from one country to another was made extremely difficult by the closure of borders and restrictions on the deployment of experts between countries. This highlighted the need to engage African's schools of public health in the Global Outbreak Alert and Response Network (GOARN) [44] to increase surge capacity in African region.

When the SARS–CoV-2 outbreak was declared a Public Health Emergency of International Concern [1], WHO did not recommend any restrictions on travel or trade. Unfortunately, as the COVID-19 pandemic spread across the region, all countries started to strictly limit international travel or completely close their borders. These restriction measures not only had a negative impact on populations and economies, but also highlighted the need for a harmonised and coordinated approach to preparedness and response to pandemics. Pre-established agreements on establishing humanitarian corridors and solidarity flights to minimise the impact of restrictions on the populations and economies are necessary. The process of making decisions on restrictive measures was taken by member states separately without clear evidence of the use of data and risk assessment. A harmonised tool for assessing the risk level at national and sub-national levels and adjusting PHSM in order to avoid nationwide movements and closure of businesses is critical for the management of the ongoing and future pandemics.

PHSM have been key to controlling the pandemic. As the pandemic evolved over time, the overall adherence level of the community towards the recommended COVID-19 preventive measures became low. Learning from experiences in responding to Ebola outbreaks and HIV, member states should consider investing more in engaging communities in responding to the pandemic. Community leaders should be turned from beneficiaries to partners for a higher buy-in of PHSM [45].

The management of data from all the pillars of the pandemic response has been sub-optimal in most countries. In the absence of quality data, it has been challenging to perform in-depth analysis to inform operational and strategic decision-making. For instance, essential public health interventions such as contact tracing were implemented sub-optimally, resulting in a minimal search for contacts lost to follow-up in the absence of quality data from contacts tracing. Building a data use culture within WHO country offices and Ministries of health should be one of the priority areas of work for WHO/AFRO in the coming years.

Finally, the circulation of new VOC has highlighted the need for strengthening member states' capacity in genomic surveillance, which should be a systemic part of the response strategies to any outbreaks with a high risk of spreading at regional and international levels.

One year after the notification of the first confirmed case in the African region, the COVID-19 pandemic remains a major public health concern, and most countries are still experiencing community transmission. Even though new reports of cases are declining in most countries, the probability of occurrence of subsequent upsurges in the African region remains high. This is for multiple reasons including but not limited to population fatigue in complying with preventive measures, the upcoming national elections in several countries leading to rallies, the possible circulation of existing SARS-CoV-2 VOC becoming predominant in some countries in addition to South Africa and the occurrence of new SARS-CoV-2 VOC that are more transmissible and cause more severe illness. WHO/AFRO has played a key role in supporting member states in their efforts to contain the pandemic and in coordinating partners' contributions. It is critical to not only learn from the past 12 months' experience to improve the effectiveness of the response to the COVID-19 pandemic but also to start preparing health systems in the African region to cope with subsequent waves of the current pandemic and future pandemics.

## Limitations

In this study, we provided data on COVID-19 cases, deaths, recoveries and health workers infections assuming that there have been no cases of re-infection, which would have led to double-counting of cases. The COVID-19 attack rate for each country was calculated based only on reported cases, which included only confirmed cases, as member states are not reporting probable cases. Given the low testing capacity in most countries, there is likely under-detection of cases leading to under-estimation of the reported incidence. Further investigations are needed to better estimate the COVID-19 incidence as seroprevalence data will be available for most member states.

The interpretation of the results presented here should take into account these limitations.

## Data Availability

The data that support the findings of this study are available on request from the corresponding author (BI). Some of the data are publicly available through situation reports produced by Ministries of Health and WHO/AFRO on their respective websites.

## References

[1] The American Journal of Managed Care. A Timeline of COVID-19 Developments in 2020. https://www.ajmc.com/view/a-timeline-of-covid19-developments-in-2020 (Accessed 17 November 2020).

[2] World Health Organization (2014) Basic Documents, 48th Edn. WHO Library Cataloguing in Publication Data, http://apps.who.int/gb/bd/PDF/bd48/basic-documents-48th-edition-en.pdf#page=1.

[3] World Health Organization. About WHO: Regional Office for Africa. https://www.who.int/about/regions/afro/en/ (Accessed 1 October 2020).

[4] Fenollar F and Mediannikov O (2018) Emerging infectious diseases in Africa in the 21st century. New Microbes and New Infections 26, S10–S18. 30402238 10.1016/j.nmni.2018.09.004 PMC6205565

[5] Wolicki SB (2016) Public health surveillance: at the core of the global health security agenda. Health Security 14, 185–188. 27314658 10.1089/hs.2016.0002 PMC6937158

[6] WHO Regional Office for Africa. COVID-19 in the African region: external situation report 1. https://apps.who.int/iris/bitstream/handle/10665/331330/SITREP_COVID-19_WHOAFRO_20200304-eng.pdf (Accessed 9 September 2020).

[7] World Health Organization Regional Office for Africa. COVID-19 in the African region: external situation report 12. (https://apps.who.int/iris/bitstream/handle/10665/332150/SITREP_COVID-19_WHOAFRO_20200520-eng.pdf (Accessed 20 January 2021).

[8] Ilesanmi OS, Bello AE and Afolabi AA (2020) COVID-19 pandemic response fatigue in Africa: causes, consequences, and counter-measures. The Pan African Medical Journal 37, 37. 10.11604/pamj.supp.2020.37.1.26742 PMC7796831 33456661

[9] World Health Organization (2016) The International Health Regulation (2005). Available at https://www.who.int/ihr/publications/9789241580496/en/ (Accessed 7 April 2019).

[10] Kasolo F (2020) IDSR as a platform for implementing IHR in African countries. Biosecurity and Bioterrorism 11, 163–169. 10.1089/bsp.2013.0032 PMC3779000 24041192

[11] World Health Organization. Public Health Surveillance for COVID-19. https://www.who.int/publications/i/item/who-2019-nCoV-surveillanceguidance-2020.8 (Accessed 6 May 2021).

[12] The World Bank. United Nations Population Division. World Population Prospects: 2019 Revision. https://data.worldbank.org/indicator/SP.POP.TOTL?locations=ZG (Accessed 17 January 2021).

[13] R Core Team. The R Project for Statistical Computing. https://www.r-project.org (Accessed 13 September 2020).

[14] The World Bank. International tourism, number of arrivals. https://data.worldbank.org/indicator/ST.INT.ARVL (Accessed 17 January 2021).

[15] Gilbert M (2020) Preparedness and vulnerability of African countries against importations of COVID-19: a modelling study. Lancet (London, England) 395, 871–877. 10.1016/S0140-6736(20)30411-6 PMC7159277 32087820

[16] Skrip LA (2021) Seeding COVID-19 across Sub-Saharan Africa: an analysis of reported importation events across 49 countries. The American Journal of Tropical Medicine and Hygiene 104, 1694–1702. 10.4269/ajtmh.20-1502 PMC8103462 33684067

[17] World Health Organization. Diabetes country profiles 2016. https://www.who.int/diabetes/country-profiles/en/ (Accessed 31 December 2020).

[18] World Health Organisation. World Health Statistics 2020. https://apps.who.int/iris/bitstream/handle/10665/332070/9789240005105-eng.pdf (Accessed 07 Januuary 2020).

[19] World Health Organization. Public health criteria to adjust public health and social measures in the context of COVID-19: annex to considerations in adjusting public health and social measures in the context of COVID-19, 12 May 2020. https://apps.who.int/iris/bitstream/handle/10665/332073/WHO-2019-nCoV-Adjusting_PH_measures-Criteria-2020.1-eng.pdf?sequence=1&isAllowed=y (Accessed 25 February 2021).

[20] Musa HH (2020) Addressing Africa's pandemic puzzle: perspectives on COVID-19 transmission and mortality in Sub-Saharan Africa. International Society for Infectious Diseases 102, 482–488. 10.1016/j.ijid.2020.09.1456 PMC7526606 33010461

[21] Usuf E and Roca A (2021) Seroprevalence surveys in Sub-Saharan Africa: what do they tell us? Lancet Global Health 9, E724–E725. 33711261 10.1016/S2214-109X(21)00092-9

[22] Mulenga LB (2021) Prevalence of SARS-CoV-2 in six districts in Zambia in July, 2020: a cross-sectional cluster sample survey. Lancet Global Health 9, e773–e781. 33711262 10.1016/S2214-109X(21)00053-X PMC8382844

[23] Chitungo I (2020) COVID-19: unpacking the low number of cases in Africa. Public Health in Practice 1, 1–2. 10.1016/j.puhip.2020.100038 PMC7485446 34173573

[24] Ghosh D, Bernstein JA and Mersha TB (2020) COVID-19 pandemic: the African paradox. Journal of Global Health 10, 1–9. 10.7189/jogh.10.020348 PMC7506193 33110546

[25] Diop ZB (2020) The relatively young and rural population may limit the spread and severity of COVID-19 in Africa: a modelling study. BMJ Global Health 5, e002699. 10.1136/bmjgh-2020-002699 PMC7252974 32451367

[26] African COVID-19 Critical Care Outcomes Study (2021) Patient care and clinical outcomes for patients with COVID-19 infection admitted to African high-care or intensive care units (ACCCOS): a multicentre, prospective, observational cohort study. Lancet (London, England) 397, 1885–1894. 10.1016/S0140-6736(21)00441-4 PMC8137309 34022988

[27] Mwananyanda L (2021) Covid-19 deaths in Africa: prospective systematic postmortem surveillance study. British Medical Journal 372, n334. 33597166 10.1136/bmj.n334 PMC7887952

[28] Adepoju P (2020) Africa's struggle with inadequate COVID-19 testing. Lancet Microbe 1, E12. 32835324 10.1016/S2666-5247(20)30014-8 PMC7212979

[29] Charvát AJ (1968) The mission of the World Health Organization. The Journal of Czech Physicians 7, 975–977. 5679073

[30] World Health Organization. Country & Technical Guidance - Coronavirus disease (COVID-19). https://www.who.int/emergencies/diseases/novel-coronavirus-2019/technical-guidance-publications (Accessed 21 March 2021).

[31] World Health Organization. Emergency Response Framework. https://www.who.int/hac/about/erf_.pdf (Accessed 31 December 2020).

[32] World Health Organization. 2019 Novel Coronavirus (2019-nCoV): Strategic preparedness and response plan. https://www.who.int/publications/i/item/strategic-preparedness-and-response-plan-for-the-new-coronavirus (Accessed 20 March 2021).

[33] Word Health Organization Regional Office for Africa. COVID-19 Strategic Response Plan in the WHO African Region. https://www.afro.who.int/publications/covid-19-strategic-response-plan-who-african-region (Accessed 21 January 2021).

[34] The World Bank. World Bank's Response to COVID-19 (Coronavirus) in Africa. https://www.worldbank.org/en/news/factsheet/2020/06/02/world-banks-response-to-covid-19-coronavirus-in-africa (Accessed 30 July 2021).

[35] African Development Bank Group. African Development Bank approves $27.33 million to ramp up the African Union's COVID-19 Response Initiative. https://www.afdb.org/en/news-and-events/press-releases/african-development-bank-approves-2733-million-ramp-african-unions-covid-19-response-initiative-37795 (Accessed 30 July 2021).

[36] Africa Center of Disease Control. COVID-19 Pandemic Response Initiatives. https://africacdc.org/download/covid-19-pandemic-response-initiatives/ (Accessed 30 July 2021).

[37] The Global Fund. COVID-19 response mechanism. https://www.theglobalfund.org/en/covid-19/response-mechanism/ (Accessed 30 July 2021).

[38] Maeda JM and Nkengasong NJ (2021) The puzzle of the COVID-19 pandemic in Africa. Science (New York, N.Y.) 371, 27–28. 10.1126/science.abf8832 33384364

[39] Umaru FA (2020) Scaling up testing for COVID-19 in Africa: responding to the pandemic in ways that strengthen health systems. African Journal of Laboratory Medicine 9, a1244. 10.4102/ajlm.v9i1.1244 PMC7276346 32537428

[40] Salyer SJ (2021) The first and second waves of the COVID-19 pandemic in Africa: a cross-sectional study. Lancet (London, England) 397, 1265–1275. 10.1016/S0140-6736(21)00632-2 PMC8046510 33773118

[41] World Health Organization. COVID-19 Weekly Epidemiological Update – 2 March 2021. https://www.who.int/publications/m/item/weekly-epidemiological-update---2-march-2021 (Accessed 20 June 2021).

[42] Gavi, the Vaccine Alliance. WHAT IS COVAX? https://www.gavi.org/covax-facility?gclid=CjwKCAjwgISIBhBfEiwALE19SRhi_lIHojqHTbcUoOgdXY197F-rZeH0C84DZlOlGY0bYO2oB1h2LxoCG5oQAvD_BwE#what (Accessed 28 July 2021).

[43] Our World in Data. Coronavirus (COVID-19 vaccinations. https://ourworldindata.org/covid-vaccinations (Accessed 08 March 2021).

[44] World Health Organization. Global Outbreak Alert and Response Network (GOARN). https://extranet.who.int/goarn/ (Accessed 28 March 2021).

[45] Benido I (2021) Preparing for COVID-19 resurgence in the WHO African region. Lancet (London, England) 397, 373. 10.1016/S0140-6736(20)32725-2 PMC7817410 33484634

